# Impact of laser modification on the contact angle and surface wettability of hydroxyapatite-coated PEEK implant material

**DOI:** 10.3389/fdmed.2026.1669867

**Published:** 2026-09-21

**Authors:** Stuti Mishra, Nayana Prabhu, Pradeep Sherigar, Veene Hegde, Vaishnavi M. Nayak, Sharon Saldanha

**Affiliations:** 1 Department of Prosthodontics and Crown & Bridge, Manipal College of Dental Sciences, Manipal Academy of Higher Education, Manipal, India; 2 Department of Prosthodontics and Crown & Bridge, Manipal College of Dental Sciences Mangalore, Manipal Academy of Higher Education, Manipal, India

**Keywords:** biomaterials, contact angle, hydroxyapatite, laser surface modification, polyetheretherketone, wettability

## Abstract

**Introduction:**

Polyetheretherketone (PEEK) is increasingly used in dental implantology because of its biomimetic properties. However, its application is limited by bio-inertness and inherent hydrophobicity, which may affect early biological interactions. PEEK surfaces are often coated with bioactive materials such as hydroxyapatite (HA), but it remains unclear how subsequent surface modification techniques such as laser treatment affect their wettability. There is still limited data on blood surface interactions in laser-treated HA/PEEK composites. This study was designed to assess and compare the blood contact angle of HA/PEEK surfaces before and after laser modification.

**Materials and methods:**

Twenty-one HA-coated PEEK discs were evaluated, with each individual disc serving as the experimental unit for contact-angle assessment. Blood contact-angle measurements were obtained from each disc before laser treatment and repeated on the same disc following laser surface modification, resulting in paired observations. Hydroxyapatite (HA) nano powder was applied to the PEEK surfaces using friction stir processing (FSP). Fresh EDTA-anticoagulated whole blood obtained from a single donor was used as the test fluid. To minimize variability, blood was collected from the same donor and venipuncture site, and all measurements were performed by the same operator under standardized conditions. Following baseline contact-angle measurement, the HA/PEEK discs were treated using an Er,Cr:YSGG laser (2,790 nm, 1 W) for 4 s at a working distance of 10 mm under 5% water and 95% air spray, after which contact-angle measurements were repeated. Pre- and post-laser contact-angle measurements were compared using the Wilcoxon signed-rank test, with statistical significance set at *p* < 0.05.

**Results:**

Pre-laser HA/PEEK surfaces exhibited lower blood contact angles than the corresponding post-laser surfaces, indicating greater wettability before laser modification. Following laser treatment, blood contact angles increased, indicating reduced wettability under the laser parameters investigated.

**Conclusion:**

The observed reduction in wettability was specific to the laser parameters evaluated in this study.

## 1 Introduction

Dental implants are widely used to replace missing teeth and restore oral function in partially or completely edentulous patients. For several decades, commercially pure titanium and titanium alloys have been the materials of choice for dental implants due to its favorable mechanical properties, low density (4.5 g/cm^3^) and complete inertness in the body environment and biocompatibility (1). These include aesthetic concerns, potential hypersensitivity reactions, elastic modulus of Ti (110 GPa) was significantly higher when compared to bone (14 GPa) leading to stress shielding and peri-implant bone overload, and susceptibility to peri-implantitis (2).

Zirconia (zirconium dioxide) implants have been developed as a metal-free substitute in response to these limitations, especially for patients with high aesthetic standards or suspected metal sensitivity.

Zirconia based materials have been claimed as a biomaterial with a high chemical stability that avoid the release of toxic products to the surrounding tissues, it provides stimulation of osteogenic cells during osseointegration in combination with unique mechanical characteristics such as high fracture toughness, fatigue resistance, high bending strength, high corrosion resistance, and radiopacity (3). Compared to Ti, early implant loss is seen due to the lack of osseointegration and therefore requires improvement by surface modification; however, concerns persist regarding its brittleness, low fracture toughness, and risk of catastrophic failure under occlusal loading, especially in posterior load-bearing regions (4). In addition, zirconia implants lack the extensive long-term clinical evidence available for titanium systems, limiting their widespread adoption (5, 6). These limitations have encouraged the exploration of alternative implant biomaterials that can combine favorable mechanical properties with improved biological performance.

Given the limitations associated with both titanium and zirconia implants, alternative biomaterials have been explored. Polyetheretherketone emerged as a promising candidate due to its favorable mechanical properties, biocompatibility, radiolucency, and elastic modulus closer to that of cortical bone, which may reduce stress shielding (7). Furthermore, its tooth-colored appearance offers aesthetic advantages in dental applications. PEEK is a high-performance polymer that belongs to the family of polyaryletherketone polymers (8–10).

To improve the osteogenic activity of PEEK, surface treatments like physical and chemical methods have been researched and developed (10).

Coating the surface with hydroxyapatite (HA), a material with identical chemical composition to human bone and compatibility has been shown to induce bone growth surrounding implants (11–13). In addition to its extraordinary capacity to bind directly to bone, HA also has osteoconductive properties (14). Consequently, hydroxyapatite is commonly applied by mixing hydroxyapatite or precursor compounds with PEEK to form bioactive composites (15). These composites have been fabricated using a range of processing techniques, for example melt mixing and injection molding plasma spraying being the most effective method. However, plasma spraying of HA is required to be carried out at a high temperature, in order to address this drawback, friction stir processing (FSP),a solid phase processing technique, has been employed to modify the surface of PEEK It was found that the FSP has been successfully to form a nanocomposite layer of HA-PEEK on the surface of the PEEK sample and effectively improved its bioactivity**.** With this technique, the substrate may be uniformly covered without having to melt it first (16). Although hydroxyapatite-modified PEEK surfaces have been investigated for improving bioactivity, most studies focus primarily on coating methods and microstructural changes. The influence of subsequent surface treatments on the wettability of HA-coated PEEK, particularly under biologically relevant conditions, remains insufficiently explored.

FSP has been proven to be a successful method for creating nanocomposites while increasing the workpiece's microstructural alteration. The creation of fine-grained structures, surface composites, and microstructural alteration of castings has all benefited from the use of this technology (17).

Immediately after placement, the implant surface is the first component to interact with blood and biological fluids, The impact of surface characteristics such as surface roughness, chemistry, wettability, and topography strongly influences the following cellular behavior. The most used techniques to measure surface wettability are contact angle measurements, which measure the interaction between biological fluids, cells, and the biomaterial surface (18). It has long been believed that osseointegration occurs from increased wettability, which is demonstrated by a low contact angle (19).

Lasers are painless and safe instruments used in dental procedures. A wide range of clinical procedures, including the etching and bleaching tooth tissues, the prevention or preparation of cavities, the removal of existing restorations, and the treatment of dentinal hypersensitivity, can be performed by lasers. By using lasers to etch dental material surfaces, the bonding quality can be improved by creating a rough texture on bonded surfaces. Although laser treatment has been shown to influence wettability and surface energy of implant materials (20), most existing studies evaluate these effects using water or saline as test liquids. Data on blood–surface interactions, especially for laser-modified HA/PEEK composites, remain limited (21).

PEEK is a hydrophobic material with a water contact angle of eighty to ninety degrees. Surface modifications are needed to change the hydrophobic nature of PEEK and lower the contact angle (22). Higher hydrophilicity in modified PEEK can lead to greater cellular proliferation. Increased cellular proliferations and hydrophilicity lead to good bone binding with the surrounding tissues. This bone binding leads to better osseointegration of the implant (23).

Although laser surface modification has been explored as a method to alter implant surface characteristics, and hydroxyapatite-reinforced PEEK composites have been widely investigated to enhance bioactivity, the combined influence of these two approaches on surface wettability has not been sufficiently studied. Most previous investigations have evaluated wettability using distilled water or saline, which may not adequately represent the biological environment encountered immediately after implant placement. The implant surface first comes into contact with blood, and these early blood–surface interactions play a crucial role in determining subsequent protein adsorption, cell attachment, and ultimately osseointegration. However, information regarding the blood wettability of laser-modified HA/PEEK surfaces remains limited. Therefore, the present study aimed to evaluate and compare the blood contact angle of HA/PEEK surfaces before and after laser modification, to better understand the early biological interactions that may influence implant biocompatibility and integration.

## 2 Materials and Methods

### 2.1 Study design and sample calculation

An *in-vitro*-repeat-measures study was conducted in the Department of Prosthodontics and Crown & Bridge, Manipal College of Dental Sciences, Manipal. Eleven parent PEEK specimens were used, from which 22 HA-coated PEEK discs were obtained. One disc was unusable and excluded, resulting in 21 discs for experimental analysis. Each individual HA-coated PEEK disc constituted the experimental unit. The study was powered at 80% with an *α* error of 5% and an effect size of 0.9. Contact-angle measurements were obtained from the same discs before and after laser surface modification, resulting in paired pre- and post-laser observations.

### 2.2 Sample design and fabrication

PEEK samples in cuboidal form (20 mm × 20 mm × 10 mm) were designed using Exocad software (Darmstadt, Germany) and milled using a VHF CAD/CAM milling machine (Germany) (Figure 1). Post milling, the surfaces were standardized using 220-grit silicon carbide abrasive paper applied with finger pressure for 10 s, followed by cleansing in 99.9% acetone for 10 s to remove surface debris.

**Figure 1:**
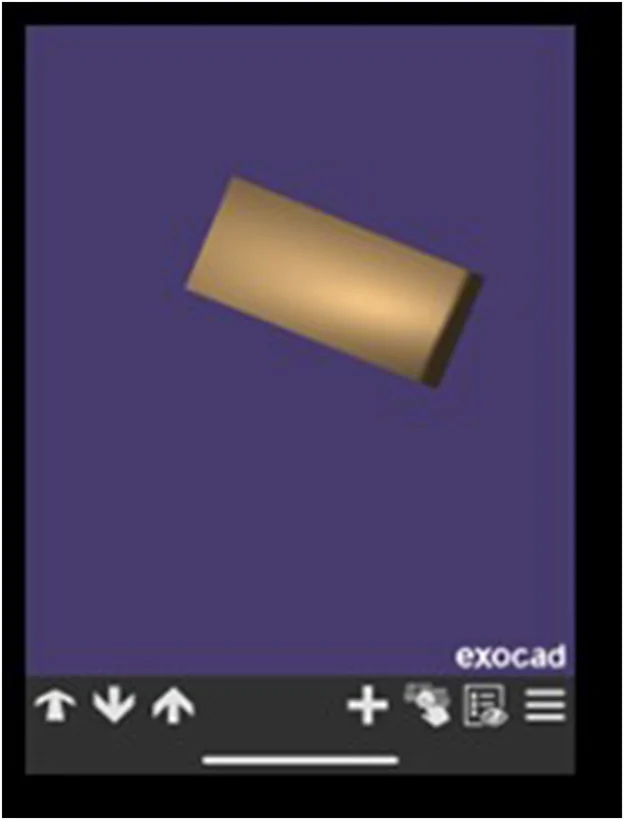
Three-dimensional CAD model of the PEEK specimen designed using exocad software for surface modification and contact angle evaluation. Exo CAD Designing.

### 2.3 Hydroxyapatite coating via friction stir processing

To facilitate uniform coating, a mold matching the sample dimensions was carved into an aluminum base plate using a vertical machining center. The samples were placed into this mold and coated with a suspension of 20 wt% hydroxyapatite (HA) nano powder (20–80 nm) in ethanol. Coating was performed using friction stir processing (FSP) with an 8 mm diameter pinned tool operating at 800 rpm for 10 s. (Figure 2) Each parent PEEK specimen yielded two HA-coated discs following friction stir processing. Each parent PEEK specimen yielded two HA-coated discs following friction stir processing. Each resulting disc was considered an individual experimental unit for subsequent contact-angle assessment. (Figure 3)

**Figure 2:**
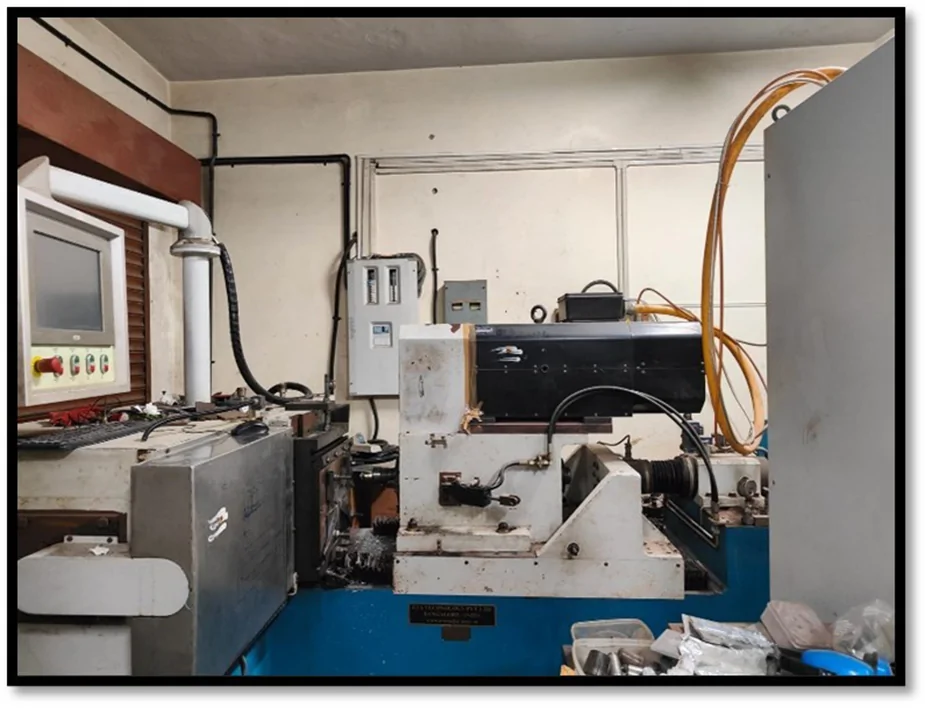
Friction stir processing setup used for hydroxyapatite coating of PEEK specimens.

**Figure 3:**
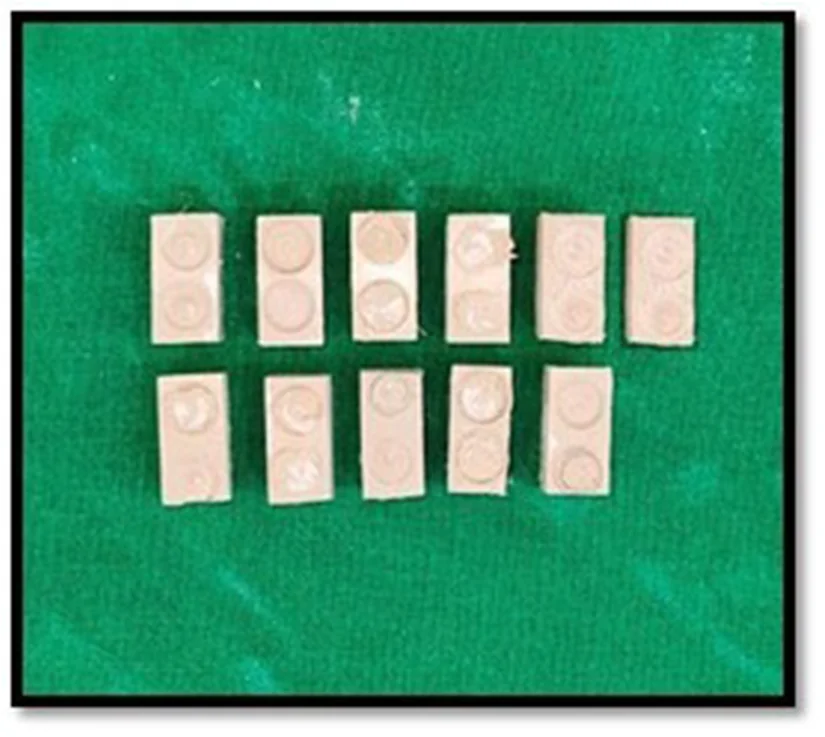
PEEK specimen after hydroxyapatite surface coating achieved by friction stir processing.

### 2.4 Laser surface modification

Following baseline contact-angle measurement, the HA-coated PEEK discs were subjected to surface treatment using an Er,Cr:YSGG laser operating at a wavelength of 2,790 nm and a power output of 1 W for 4 s. The laser was delivered as a continuous beam with a Gaussian energy profile, with the fiber tip maintained at a fixed working distance of 10 mm from the specimen surface. The laser tip was moved in a controlled sweeping motion across the specimen to ensure uniform irradiation of the coated area. A water–air spray (5% water, 95% air) was applied during irradiation to prevent thermal damage and maintain surface stability during laser exposure. (Figure 4) These parameters were selected to balance effective surface modification with preservation of coating integrity and substrate stability and were consistent with previously reported laser settings used to modulate surface roughness and topography without causing deleterious surface damage (24, 25).

**Figure 4:**
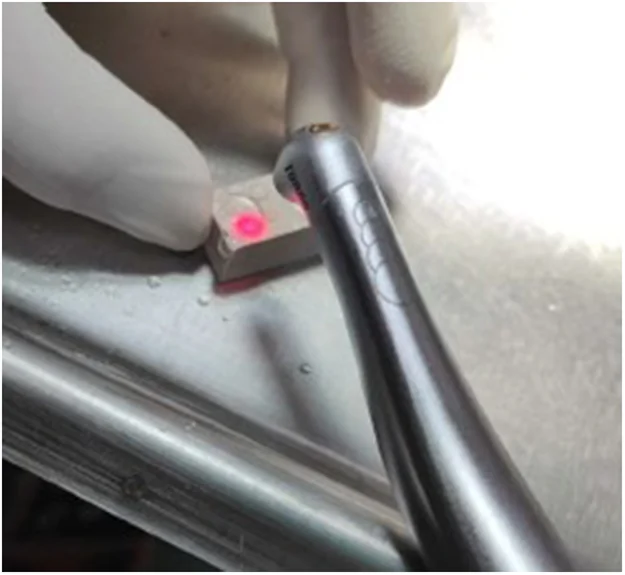
Er,Cr:YSGG laser irradiation of the hydroxyapatite-coated PEEK specimen during surface modification.

Unreacted surface salts were carefully removed by using a soft bristle brush to ensure a clean and uniform surface while preserving the integrity of the laser-modified surface morphology. No rinsing or chemical cleaning was performed post-laser treatment, as such procedures could alter the physicochemical characteristics intended for evaluation. No polishing was performed post laser treatment to preserve the laser-induced surface modifications. The rationale for this decision was to maintain the surface modifications induced by the Er,Cr:YSGG laser, which were critical for assessing changes in wettability. However, prior to coating and laser treatment, all samples were standardized using 220-grit silicon carbide abrasive paper to ensure uniform baseline conditions across specimens.

Contact angle measurements were first recorded to determine baseline wettability prior to laser treatment and were repeated after laser surface modification. The pre-laser measurements served as the baseline/control condition for comparison with the post-laser measurements obtained from the same discs. Laser application was intended to induce surface-level morphological and physicochemical modification of the hydroxyapatite-coated surface rather than bulk material modification or deep penetration into the PEEK substrate. No chemical synthesis or phase transformation was assumed beyond surface modification.

### 2.5 Blood collection and contact angle measurement

Approximately 3 mL of fresh whole blood was collected aseptically from the median cubital vein of a single healthy donor (primary investigator) into EDTA (lavender-top) anticoagulant tubes. To minimize variability, all measurements were performed using blood obtained from the same donor, collected from the same venipuncture site, and handled by the same operator under standardized conditions. A single blood droplet was placed at the center of each sample. The droplet was dispensed using a calibrated micropipette delivering approximately 5 µL of blood to ensure consistent droplet volume across all specimens. Following the baseline contact-angle measurement, residual blood was removed from the specimen surface using ethanol, and the specimen was dried before laser irradiation. Following laser treatment, a fresh blood droplet was applied for post-laser contact-angle measurement. Contact-angle measurements were obtained from each specimen before laser modification and repeated on the same specimen following laser treatment. The contact angles were measured at two different points using the FTA200 contact angle analyzer. (Figure 5) It is a flexible video system used to measure the contact angle. The camera used in the device can capture images from 1/60 s to hours. Measurements were conducted at room temperature (approximately 25 °C) under ambient laboratory humidity. The dimension of the blood droplet was smaller than the area of the laser modification, and the blood droplet was kept in the center of the specimen. The Gaussian beam produces the maximum energy in the center of the specimen; hence, the blood drop was kept in the center of the specimen.

**Figure 5:**
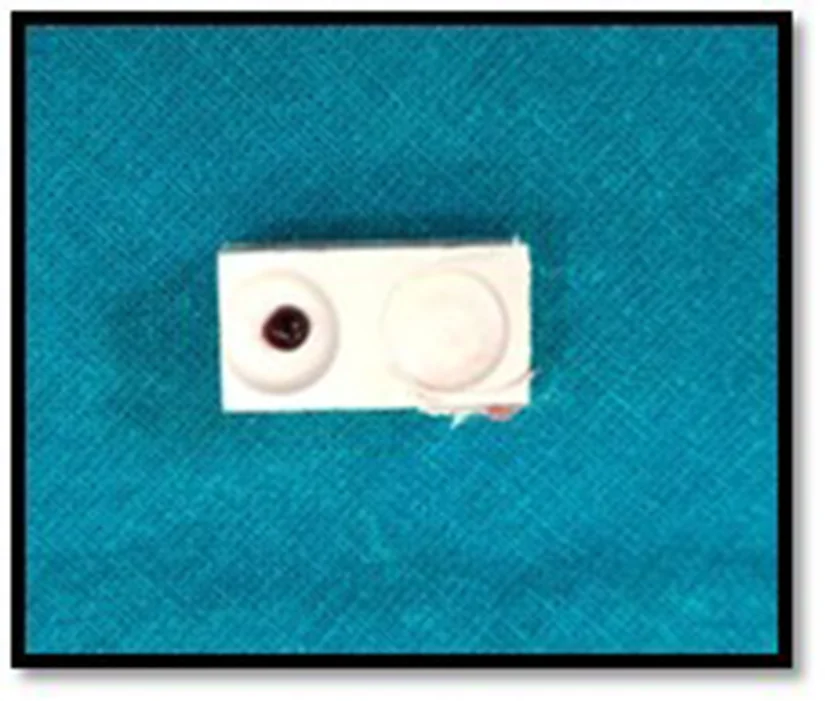
Placement of a blood droplet on the hydroxyapatite-coated PEEK surface for wettability assessment.

The system finds the points where the baseline and drop profile intersect to measure the contact angle. The contact angle is the difference between the baseline and drop profile angles. (Figure 6). The contact angle was measured at the right and left baseline intersections of the droplet profile, designated as Points A and B, respectively. Both measurements were recorded to assess potential asymmetry in droplet–surface interaction.

**Figure 6:**
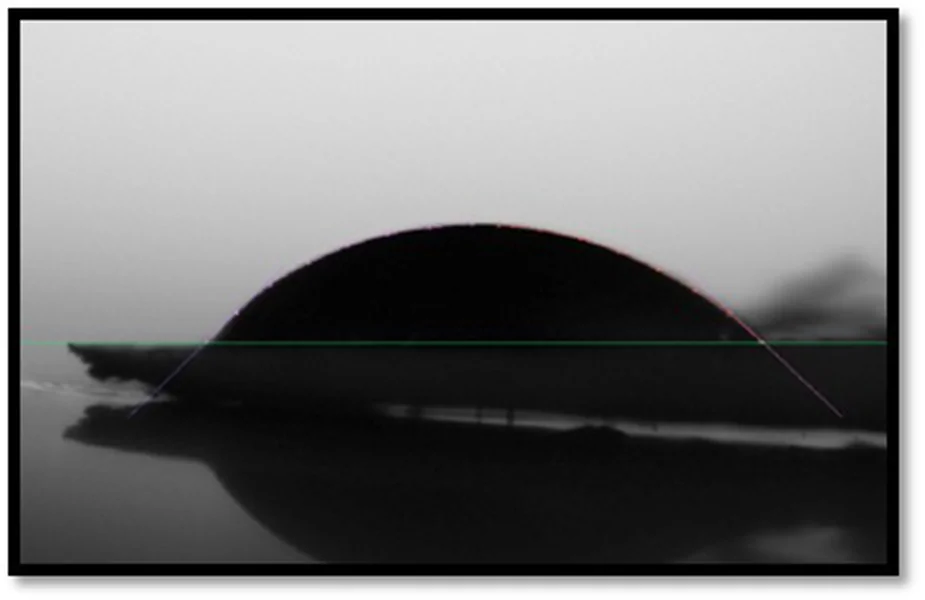
Measurement of blood contact angles on the HA/PEEK surface using a contact angle analyzer.

The drop was placed on the HA/PEEK surface and allowed to stabilize for approximately 10 s before digital contact angle measurement to minimize variations caused by early protein adsorption or coagulation. All laser surface modifications were performed on dry, HA-coated PEEK specimens under controlled water–air spray cooling, in accordance with the laser operating protocol. Blood was introduced only during contact angle measurement to assess wettability and early biological fluid–surface interaction relevant to implant integration.

### 2.6 Statistical analysis

Data was analyzed using SPSS version 26 (SPSS Inc., Chicago, IL). Normality was assessed using the Shapiro–Wilk test. As the data did not consistently satisfy the assumption of normality, non-parametric statistical tests were used. Paired comparisons between pre-laser and post-laser contact-angle measurements obtained from the same specimens were performed using the Wilcoxon signed-rank test. Comparisons between Points A and B within each experimental condition were also performed using the Wilcoxon signed-rank test, as these measurements were obtained from the same specimens. A *p*-value < 0.05 was considered statistically significant.

## 3 Results

The study evaluated the effect of laser surface modification on the blood contact angle of HA/PEEK composite specimens by comparing measurements obtained pre and post laser treatment. Blood contact angles were significantly higher following laser modification, indicating reduced wettability.

At Point A, the median blood contact angle increased from 32.74° (IQR: 46.18°) pre laser treatment to 76.34° (IQR: 13.82°) a post laser treatment. The corresponding mean ± SD values increased from 46.09 ± 23.78° to 79.15 ± 10.10°, respectively. The Wilcoxon signed-rank test demonstrated a statistically significant difference between the paired pre- and post-laser measurements (Z = 3.360, *p* < 0.001) (Table 1). A box plot (Figure 7) illustrates the distribution of Point A contact-angle measurements before and after laser modification.

**Table 1:** Comparison of blood contact angle at Point A pre and post laser modification.

Statistic	Pre laser treatment	Post laser treatment
Median (IQR),°	32.74 (46.18)	76.34 (13.82)
Mean ± SD,°	46.09 ± 23.78	79.15 ± 10.10
Minimum,°	15.45	60.68
Maximum,°	85.40	99.65
Wilcoxon Z	**3** **.** **360**	
*p*-value	**0**.**001**	

Values are presented as median (interquartile range [IQR]) and mean ± standard deviation (SD). Paired pre- and post-laser measurements were compared using the Wilcoxon signed-rank test. Bold values indicate a statistically significant difference (*p* < 0.05).

**Figure 7:**
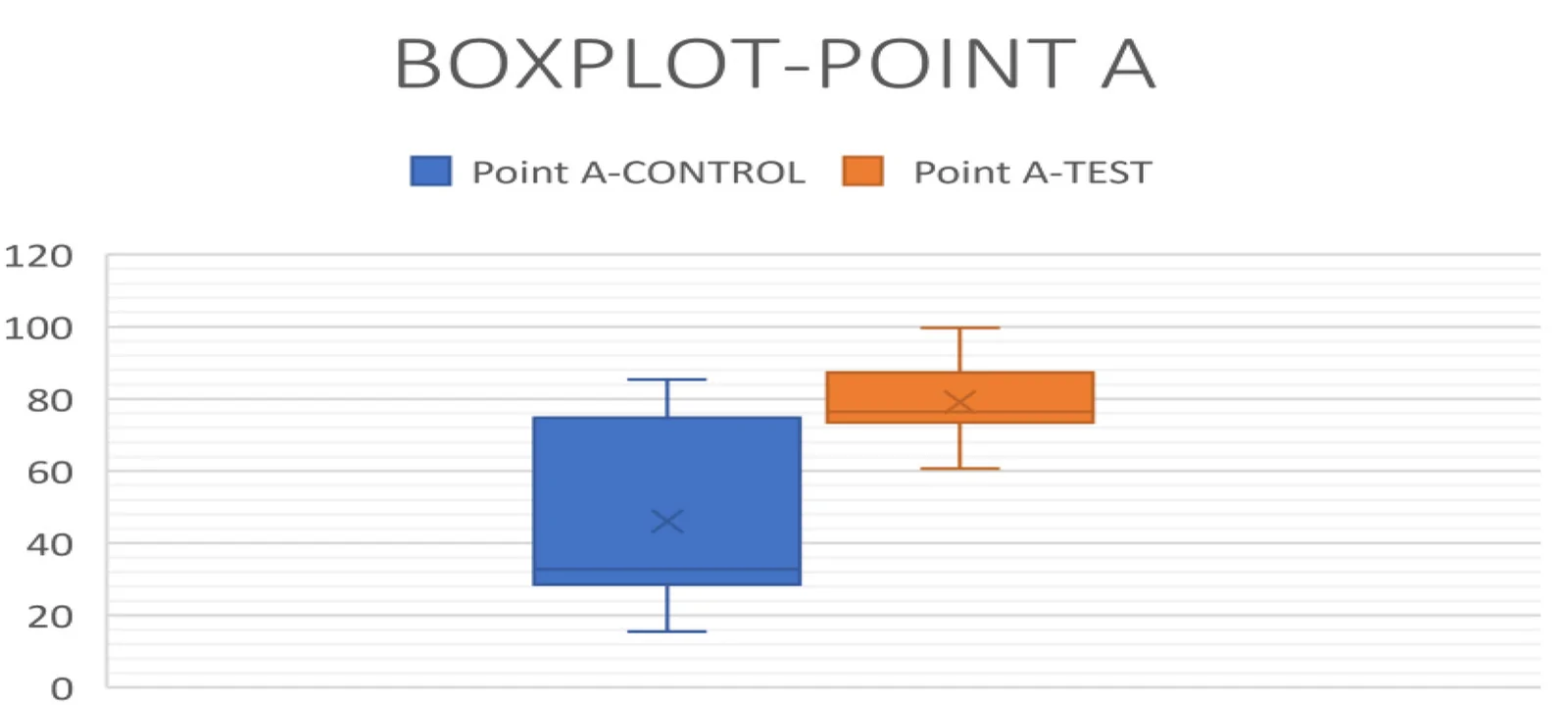
Box plot showing the distribution of contact-angle measurements at Point A under the pre-laser and post-laser conditions.

At Point B, the median blood contact angle increased from 37.64° (IQR: 47.26°) pre laser treatment to 80.89° (IQR: 7.92°) post laser treatment. The corresponding mean ± SD values increased from 44.51 ± 23.62° to 82.06 ± 7.46°, respectively. The Wilcoxon signed-rank test demonstrated a statistically significant difference between the paired pre- and post-laser measurements (Z = 3.597, *p* < 0.001) (Table 2). A box plot (Figure 8) illustrates the distribution of Point B contact-angle measurements before and after laser modification.

**Table 2:** Comparison of blood contact angle at Point B pre and post laser modification.

Statistic	Pre laser treatment	Post laser treatment
Median (IQR),°	37.64 (47.26)	80.89 (7.92)
Mean ± SD,°	44.51 ± 23.62	82.06 ± 7.46
Minimum,°	17.30	67.91
Maximum,°	85.01	98.46
Wilcoxon Z	3.597	
*p*-value	< 0.001	

Values are presented as median (interquartile range [IQR]) and mean ± standard deviation (SD). Paired before- and after-laser measurements were compared using the Wilcoxon signed-rank test. Statistical significance was set at *p* < 0.05.

**Figure 8:**
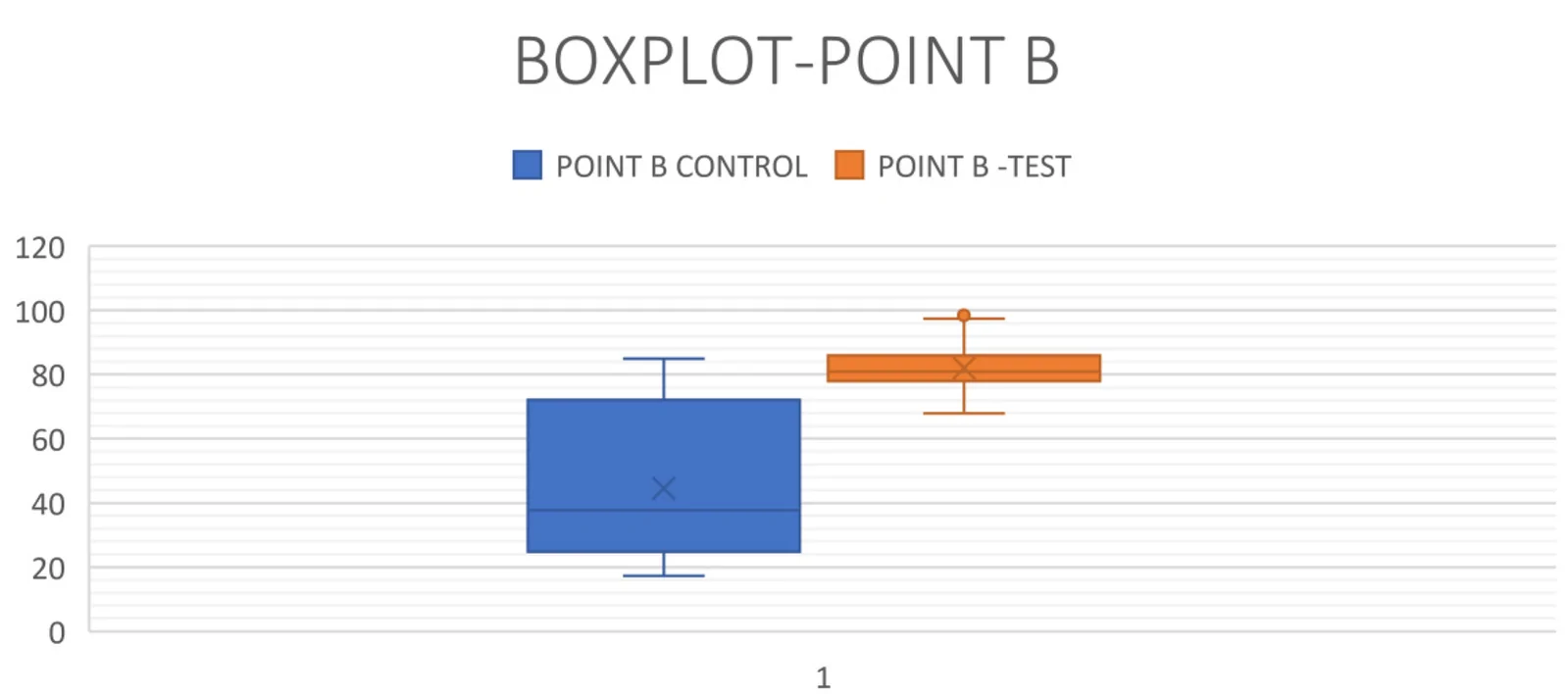
Box plot showing the distribution of contact-angle measurements at Point B under the pre-laser and post-laser conditions.

Within-condition comparisons between paired Point A and Point B measurements were performed using the Wilcoxon signed-rank test. Pre laser treatment, the median contact angles were 32.74° (IQR: 46.18°) at Point A and 37.64° (IQR: 47.26°) at Point B, with no statistically significant difference between the two measurement points (Z = −0.852, *p* = 0.394) (Table 3). Following laser treatment, the median contact angles were 76.34° (IQR: 13.82°) at Point A and 80.89° (IQR: 7.92°) at Point B, with a statistically significant difference between the two measurement points (Z = −2.972, *p* = 0.003) (Table 4).

**Table 3:** Comparison of blood contact angle between Points A and B under the pre-laser condition.

Statistic	Point A	Point B
Median (IQR),°	32.74 (46.18)	37.64 (47.26)
Mean ± SD,°	46.09 ± 23.78	44.51 ± 23.62
Wilcoxon Z	−0.852	
*p*-value	0.394	

Within-condition comparison between Points A and B was performed using the Wilcoxon signed-rank test.

Statistical significance was set at *p* < 0.05.

**Table 4:** Comparison of blood contact angle between Points A and B under the post-laser condition.

Statistic	Point A	Point B
Median (IQR),°	76.34 (13.82)	80.89 (7.92)
Mean ± SD,°	79.15 ± 10.10	82.06 ± 7.46
Wilcoxon Z	−2.972	
*p*-value	0.003	

Within-condition comparison between Points A and B was performed using the Wilcoxon signed-rank test.

Statistical significance was set at *p* < 0.05.

Overall, laser surface modification resulted in a statistically significant increase in blood contact angle at both measurement points, indicating reduced wettability of the HA/PEEK surfaces under the laser parameters investigated.

## 4 Discussion

The purpose of this study was to assess changes in blood contact angle and wettability of hydroxyapatite (HA)-coated polyetheretherketone (PEEK) surfaces before and after Er,Cr:YSGG laser modification. Blood was selected as the test fluid to simulate the biological fluid–surface interaction occurring immediately after implant placement.

The present findings demonstrated a statistically significant increase in blood contact angle following laser modification at both measurement points, indicating reduced wettability under the laser parameters investigated. Because all specimens underwent HA coating using FSP, the independent effect of FSP-mediated HA coating on wettability could not be determined from the present study.

An additional finding was that the contact angle measurements at Points A and B differed significantly under the post-laser condition, whereas no significant difference was observed between the two measurement points under the pre-laser condition. Because Points A and B represent measurements obtained from opposite sides of the blood droplet, this difference may reflect greater asymmetry in droplet–surface interaction following laser modification. However, in the absence of direct surface topography and roughness characterization, the underlying cause of this variation cannot be established and should therefore be interpreted cautiously.

### 4.1 HA coating on PEEK

Hydroxyapatite coating has been reported in previous studies to influence surface wettability through the presence of calcium- and phosphate-rich groups, which may increase surface hydrophilicity. Previous studies have suggested that such surfaces may favor protein adsorption and osteoblast adhesion (26, 27). Hydroxyapatite is widely used as an implant coating because of its hydrophilicity, bioactivity, and biocompatibility, characteristics that have been associated with enhanced bone cell attachment in previous studies (27).

### 4.2 Rationale for using FSP

Friction stir processing (FSP) is an effective solid-state processing technique used to fabricate surface nanocomposites and modify the microstructure of the substrate. The process generates significant plastic deformation and dynamic recrystallization of the material in the agitated region, resulting in the modification of its microstructure. FSP-treated surfaces have been reported to exhibit increased wettability, which may contribute to favorable biological interactions reported in previous studies (17, 28).

The ability of FSP to incorporate HA uniformly onto PEEK surfaces has been previously reported (11). Although previous studies have reported changes in wettability following FSP-mediated incorporation of HA onto PEEK surfaces, the present study did not include an uncoated PEEK control. Therefore, the independent contribution of FSP or HA coating to surface wettability cannot be determined from the present findings.

### 4.3 Interpretation of wettability changes following laser modification

According to Wenzel's model, surface roughness amplifies the intrinsic wetting characteristics of a material rather than independently determining its wettability (29). Thus, a hydrophilic surface becomes more hydrophilic, whereas a hydrophobic surface becomes more hydrophobic with increasing roughness. Because surface roughness and surface chemistry were not directly characterized in the present study, it could not be determined whether the observed increase in blood contact angle resulted from changes in intrinsic wettability or laser-induced alterations in surface topography. Previous studies have reported that incorporation of hydroxyapatite may increase the intrinsic hydrophilicity of PEEK surfaces. Previous studies on FSP-processed HA composites have reported reduced contact angles following surface modification, although the underlying physicochemical mechanisms were not evaluated in the present study (30). Previous investigations have suggested that improved wettability may favour early biological interactions; however, these outcomes were not directly evaluated in the present study (26). However, these effects are highly dependent on processing conditions. Deviations in surface modification parameters, particularly during laser treatment, may alter surface chemistry and topography in ways that do not necessarily translate into increased wettability.

### 4.4 Impact of laser modification on contact angle

The laser-powder interaction is a multifaceted phenomenon that takes place on microscopic and fast time scales, frequently on the laser beam's focal plane, which has the best power density for melting and sintering operations. Operationalizing on these tiny temporal and spatial scales implies that slight variations from the ideal processing parameters can have a substantial effect on the mechanical and structural qualities of the final manufactured parts (31). Studies have reported that laser treatment of PEEK may induce surface restructuring, including oxidation, localized melting, or partial polymer degradation, depending on the irradiation parameters. Such changes may reduce the availability of hydrophilic functional groups, including polar ketone groups, at the material surface. Previous studies have demonstrated that the influence of laser treatment on PEEK wettability is highly dependent on the irradiation parameters employed. Li et al. reported that microsecond Nd: YAG laser treatment increased the water contact angle of PEEK/CF, whereas nanosecond laser treatment reduced the contact angle by generating oxygen-containing functional groups on the surface. Similarly, the present study demonstrated a statistically significant increase in blood contact angle following Er,Cr:YSGG laser treatment at both measurement points. The differences among studies are likely attributable to variations in laser wavelength, pulse characteristics, substrate composition, surface treatment protocols, and the use of hydroxyapatite-coated PEEK rather than uncoated PEEK (32).

However, because surface topography was not characterized in the present study, this explanation remains speculative (33). A key component of PEEK's interactions with water and other biological fluids is the ketone group, which is well-known for its electron-withdrawing capabilities. Changes in surface chemistry could potentially influence the exposure of functional groups involved in hydrogen bonding (34); however, such alterations were not directly evaluated in the present study.

An alternative explanation for the increased contact angle is provided by the Cassie–Baxter wetting model, in which air becomes entrapped between the liquid droplet and the textured surface, thereby reducing the effective solid–liquid contact area and increasing the apparent contact angle. Fareed et al. demonstrated a similar increase in contact angle on DLIP-functionalized PEEK coatings and attributed this behaviour to air entrapment within the laser-generated surface microstructures. Although a similar mechanism may have contributed to the present findings, the absence of complementary surface characterization techniques such as SEM, profilometry, or chemical analysis precludes confirmation of this mechanism in the current study (35, 36).

Although DLIP-based methods have shown accurate control over surface topography to enhance biological responses, our findings indicate that laser-induced changes in wettability are parameter-dependent, highlighting the importance of optimizing laser parameters (37).

An increase in contact angle indicates reduced wettability and may influence early biological interactions reported in previous studies. However, the biological consequences of the present findings were not directly investigated. Whether the observed reduction in wettability is advantageous or detrimental for implant performance remains uncertain, as the present study did not evaluate subsequent protein adsorption, blood clot formation, cell attachment, or osseointegration.

### 4.5 Limitations

Advanced surface characterization techniques that could have provided detailed information on surface topography, roughness, and chemical composition following laser treatment, such as scanning electron microscopy (SEM), atomic force microscopy (AFM), surface profilometry, and x-ray photoelectron spectroscopy (XPS), were not employed. Consequently, the exact surface morphological or chemical changes responsible for the observed differences in wettability could not be determined, and the proposed mechanisms underlying these changes remain speculative.

In addition, direct biological assessments, such as protein adsorption studies, cell-based assays, or simulated body fluid immersion tests, were not performed. Therefore, the biological implications of the observed changes in blood contact angle, including their influence on early cell attachment and osseointegration, could not be directly established.

Furthermore, because all specimens underwent hydroxyapatite coating using friction stir processing (FSP) prior to laser treatment, the independent contributions of HA coating, FSP, and laser irradiation to the observed changes in wettability could not be evaluated separately.

Finally, although dynamic contact angle analysis over extended periods may provide additional insight into time-dependent wetting behavior, the present investigation was limited to static blood contact angle measurements to evaluate early-stage wettability relevant to the initial biological response at the implant interface. Furthermore, the significant difference between the two contact-angle measurement points in the laser-modified group suggests potential variability in droplet–surface interaction; however, the present study was not designed to determine the morphological or physicochemical basis of this variation.

## 5 Conclusion

This study evaluated changes in blood wettability of HA-coated PEEK surfaces before and after Er,Cr:YSGG laser surface modification. Blood contact angles increased significantly following laser treatment, indicating reduced wettability under the laser parameters investigated. Because all specimens underwent HA coating using FSP, the independent effects of FSP and HA coating on surface wettability could not be determined. These findings indicate that Er,Cr:YSGG laser treatment altered the blood wettability of HA/PEEK surfaces under the specific irradiation parameters evaluated. Further investigations incorporating comprehensive surface characterization and biological evaluation are required to elucidate the mechanisms underlying the observed changes in wettability and their relevance to implant performance.

### 5.1 Clinical implications

The present findings demonstrate that Er,Cr:YSGG laser treatment altered the blood wettability of FSP-mediated HA-coated PEEK surfaces under the parameters investigated. These observations may assist in optimizing laser surface modification strategies for future HA/PEEK implant applications. However, the independent effects of FSP and HA coating require evaluation using appropriate uncoated PEEK pre-laser condition in future studies.

### 5.2 Future directions

Future studies should focus on developing PEEK-based implants with optimized surface characteristics through refined laser processing protocols while preserving favorable wettability and evaluating their biological performance through *in vitro* and *in vivo* investigations.

## Data Availability

The original contributions presented in the study are included in the article/Supplementary Material, further inquiries can be directed to the corresponding author.
